# Recent Developments in Public Health Nursing in the Americas

**DOI:** 10.3390/ijerph7030729

**Published:** 2010-02-26

**Authors:** Gustavo Nigenda, Laura Magaña-Valladares, Kelly Cooper, Jose Arturo Ruiz-Larios

**Affiliations:** National Institute of Public Health (Instituto Nacional de Salud Pública), Avenida Universidad No. 655, Colonia Santa María Ahuacatitlan, C. P. 62100, Cuernavaca, Morelos, México; E-Mails: lmagana@insp.mx (L.M.V.); kellycooper3@gmail.com (K.C.); jaruiz@correo.insp.mx (J.A.R.L.)

**Keywords:** public health nursing, nursing, public health, the Americas, Latin America, essential functions of public health

## Abstract

This study presents an assessment of the participation and training of nurses in public health areas in the Americas. Information was gathered through a literature review and interviews with key informants from Mexico, Colombia, and Paraguay. Results demonstrate that there is significant variation in definitions of public health nursing across the region and current systematized data about the workforce profile of public health nursing personnel is not available for many countries in the Americas. There are significant regional differences in the levels and types of training of nurses working in public health areas and an increasing number of nurses are pursuing training in public health at the master’s and doctoral levels. Many nurses carry out some or all of the essential functions of public health, but are not considered to be public health nurses. Generally, auxiliary and technical nurses have a broader presence in public health areas than professional nurses. In the future, regional health systems reforms should support increased recruitment and training of public health nurses, as well as stronger roles in public health research and health care at the individual, community, and population levels.

## 1. Introduction

Public health nurses play important roles in health promotion and disease prevention and control at the community and population levels. Although nursing job responsibilities are often publicly perceived to be limited to direct patient care, public health nurses make substantial contributions to population health on a daily basis through their participation in patient education, disease surveillance, and public health campaigns. These contributions to public health are especially important in light of recent demographic and epidemiological shifts, health sector reforms, and health workforce changes across the Americas.

Public health nursing is a specialized field within the nursing profession because it is population-focused, as practitioners often work with community members who are not in hospitals or other health care institutions, and community-focused, as practitioners focus on the relationship between population health and the environment—which includes physical, biological, and socio-cultural factors—in order to meet public health needs [1]. Public health nurses are concerned not only with direct patient care, but also with community and population health promotion and disease prevention activities, which require an interdisciplinary focus on nursing, public health, and social sciences [2–4]. Public health nurses work in diverse settings with a variety of health actors and offer a wide range of services to patients and community members, including education and epidemiological research [5]. Despite the long history of the field of public health nursing and efforts to define its scope and practice [6], it is difficult to find systematized information about the public health nursing workforce in the Americas.

Public health nurses play important roles in promoting public health across the Americas and currently comprise the largest group of professionals in the field of public health [7]. There are significant differences in the number, job descriptions, and work responsibilities of these health workers. Furthermore, there are significant differences in the levels and types of training and education of public health nurses. Regional differences in characterizations of public health nursing are complicated by the fact that many nurses carry out some or all of the essential functions of public health, but are not considered to be public health nurses and/or are not familiar with the field of public health. It is important to note that there is a scarcity of health workers, especially nurses, across the Americas [8]. The Pan American Health Organization (PAHO) states that 15 countries in the Americas do not meet the baseline of human resource density, which is 25 health workers per 10,000 people, and emphasizes the extreme lack of nurses who are needed to maintain population health [9]. Although PAHO stated in 2001 that more research was needed to ascertain the number and composition of the public health nursing workforce, information remains difficult to find or nonexistent for many countries in the Americas [8]. According to the available data about nursing, there are high numbers of assistants and auxiliary nurses who are responsible for direct contact with patients in primary care settings [10]. There are low numbers of nurses who have advanced degrees and/or are trained for administrative or research positions. Even though some nurses have higher levels of training, many carry out the same roles as nurses with lower levels of training.

Additionally, the migration of health professionals in the Americas exacerbates existing health system inequities and workforce shortages across the Americas. The tendency of highly skilled workers to migrate increased in the 1990s due to comparative pay levels and working conditions. Currently, migration continues to strengthen health systems in developed countries, while weakening those in low- and middle-income countries. In order to meet the health systems goals of countries in the Americas, more research is needed to understand the migration of health workers, especially public health nurses [10].

## 2. Methods

This study seeks to analyze public health nursing in the Americas in regard to workforce profile, specifically education level, job responsibilities, professional roles, and the number of personnel in each country. A literature review of articles published in the past 11 years was performed to assess recent trends in public health nursing in the region. To complement the information gathered through the literature review, interviews were performed with nurses who have undertaken leadership roles in public health activities in Mexico, Colombia, and Paraguay, in order to understand the situation of public health nursing in those countries. Information from the selected countries is important for this study because in each country the field of nursing is undergoing noteworthy changes, such as increased recognition of nurses as health professionals and renewed interest in community-based nursing practice. The literature review concentrated on information about the field of public health nursing and the interviews focused on the profile of the current workforce. The literature review and interviews were performed separately. The main question used for the literature review was: Is public health nursing a recognized and defined concept and/or field in the regional literature? This general question was focused through a series of steps in order to identify keywords that relate to education level, job responsibilities, and professional roles (Table 1). These keywords were then used for the literature review strategy.

The document search was carried out in multiple steps. First, searches were performed for the keywords “public health nursing” and “the Americas,” and “*enfermería, salud pública*” and “*Las Américas*.” Next, searches were performed for “public health nursing” in combination with one geographic keyword (“Latin America,” “United States,” and “Canada”) and one English keyword from steps 3−5 of the process to identify keywords. Searches were carried out in the same way for “*enfermería, salud pública*” in combination with the Spanish keywords. All searches were performed using the Boolean operations “and” and “or.” All searches were executed by selecting the field “all text,” in order to maximize the number of articles retrieved. Then, the search was refined by selecting the field “abstract”.

Databases were selected based on the criteria that they contain information in English and Spanish, have nursing and public health journals, and provide information about the fields of public health, nursing, and education. Databases that contain Spanish and English literature were consulted in order to minimize language bias and ensure a geographically broad search for studies about public health nursing. PUBMED, which is a free search engine maintained by the U.S. National Library of Medicine, was consulted because it contains 757 journals about nursing and 315 about public health. Another free search engine that was consulted was the Virtual Health Library, which is maintained by BIREME, a Specialized Center of the Pan American Health Organization. The Virtual Health Library was consulted because it incorporates a large number of materials in Spanish about public health and contains the health sciences databases LILACS and SciELO. Additionally, EBSCO was consulted because it has 1,059 journals about nursing, 542 about public health, and 1 about public health nursing; this database included Academic Search Premier, ERIC, MEDLINE and MedicLatina. The following inclusion and exclusion criteria were established to determine article relevance:

*Inclusion criteria*
  The article:
    1. provided an interpretation and/or current definition of public health nursing in the region, or in specific countries in the region;
    2. described the relationship of public health nursing to the fields of public health and nursing;
    3. discussed the workforce profile and professional roles and/or performance of public health nurses or nurses who work in public health areas;
    4. covered the themes of education and/or competencies for public health nurses.
*Exclusion criteria*
  The article:
    1. discussed specific functions of public health nurses without providing an interpretation or definition of the field;
    2. referenced specific time periods in the history of public health without substantial reference to the current state of public health nursing;
    3. focused on individual testimonies of public health nurses, without substantial reference to the current state of public health nursing;
    4. described the socioeconomic circumstances of current public health nurses;
    5. discussed ethics in nursing, as these arguments fall outside the scope of this research.

Using the inclusion and exclusion criteria, articles relevant to the topic were selected based on the publication title and abstract content. This group of articles was reviewed for relevancy to the main question. Articles that were not relevant were eliminated. For each of the selected articles, information was extracted regarding the country or region of focus and the central themes. Then, that information was summarized in order to describe trends in public health nursing across the Americas.

Because there is little information available regarding the number of public health nursing personnel in the Americas, information was gathered about the total number of public health and nursing personnel using materials published by the United Nations Population Fund and the World Health Organization.

To complement the information gathered through the literature review, interviews were performed to determine the public health nursing workforce profile in the three chosen countries. A semi-structured interview was performed by telephone with one key informant from Colombia. Semi-structured interviews were held in person with key informants in Mexico and Paraguay. Key informants were nurses who were selected for the interviews based on their work experience, leadership in the field of nursing, expertise about recent developments in public health nursing, and participation in academia, the health sector, or both. For Colombia, an interview was conducted with a Colombian researcher at the University of Antioquia, Faculty of Public Health, who is an expert in the areas of nursing and public health. For Mexico, the Director of the Nursing Commission of the Secretary of Health was interviewed. For Paraguay, an interview was conducted with a group of seven senior-level administrators from the National University of Asuncion, the Ministry of Public Health and Social Welfare, the Paraguayan Nursing Association, and the National Institute of Continuing Education in Nursing and Obstetrics. During the interviews, the individuals interviewed provided official documents and grey literature from their respective countries.

The interviews covered the following thematic areas: the number of available nursing personnel in the country; the various categories of nursing personnel; the educational requirements and job responsibilities of nurses who work in public health areas; and the roles nurses play in public health. A guide was used to conduct the interviews (Appendix).

The interviews were tape recorded with the consent of the informants. The material was transcribed and processed using ATLAS.ti, a computer software program designed for qualitative data analysis. Using this program, codes (or concepts) were defined and organized into families consisting of related codes, then compared with other families. The information was synthesized according to the central topics this research. After the interviews were processed, information was extracted based on the criteria that it provided an analysis of public health nursing and described the status of nursing personnel in relation to public health areas, specifically in terms of the number of available nurses and their training and job performance. This information was used to develop a case study for each country.

## 3. Background

### 3.1. Public Health Concepts

Public health has been defined in several ways and over the course of its development has come to refer to a group of values and social activities focused on improving the health of the population. The concept of public health evolved from the area of hygiene, following the principle that specific actions can be taken to eliminate and/or establish barriers for infectious or pathogenic agents. The concept of public health has been modernized due to epidemiological changes in populations across the world and the emergence of a new disease profile that consists of chronic, non-infectious, and degenerative health problems.

Currently, a key element of the concept of public health is its focus on the health needs of populations rather than individuals, in contrast to direct patient care which usually refers to a clinical focus. Public health experts focus on different specific aspects of the concept. In his definition of public health, Terris highlights community participation but does not explicitly include the participation of governmental institutions [11]. In contrast, Frenk states that society and governmental institutions should assume responsibility for social welfare, which includes the achievement of better health conditions [12]. PAHO also considers the role of governmental institutions in the achievement of better health conditions of populations through health promotion, disease prevention, the diagnosis and treatment of illnesses, and rehabilitation [13].

A recent proposal supported by international health organizations states that public health can be structured in 11 essential functions, which should be carried out primarily by governmental institutions (see Table 1) [14].

It is important to note that the proper performance of the essential functions of public health requires the coordination of multiple social actors in the government, public, and private sectors. Also, the essential functions suggest that training in public health should include competencies that address a wide range of public health activities—from clinical procedures to administrative and planning tasks—which today require a stronger global perspective than initially thought when the original definitions were coined.

### 3.2. Nursing Issues in the Americas

Based on the essential functions of public health, it is possible to identify the roles that nurses should perform in health systems. It is widely recognized that nurses in Latin America have made great advances to improve their levels of training and labor conditions. However, in some countries there are major challenges for developments in the field of nursing. Analysts argue that the subordinate role of nurses in relation to doctors and other specialists within health sector managerial strategies and organizational structures restricts nurses’ ability to make clinical decisions. Consequently, this negatively impacts public perceptions about their contributions to public health and health care, including primary care and population health [15]. In countries such as Mexico this situation is highly prevalent.

Nursing in Latin America is a women-dominated profession. Although men have recently shown interest in participating in nursing training, in general, women represent between 85–95% of all nurses [16]. Furthermore, there is a regional trend to increase the number of years of training required to enter the field, but only in few countries nursing has reached the level of training of other health professions, such as medicine or dentistry, in which large proportions of practitioners have completed postgraduate studies. In many countries, nurses with basic training (between three and nine years of formal education) represent the majority of nurses working in health units. Only Brazil and Colombia have developed doctoral programs specifically for nursing.

Traditionally, nursing does not possess the prestige of other professions in health systems across the Americas. Consequently, the demand for training in nursing is not as high as it is for other fields, such as medicine. This has generated a lack of properly trained nurses and a surplus of nurses who do not meet the requirements of public and private health institutions. In most countries, efforts have been made to increase enrollment in nursing schools with differing levels of success [17].

In addition to problems with the level of nursing training, the migration of nurses, especially from Latin America countries, has impacted the profile of health human resources at the global level. Uruguay, Argentina, Peru, Colombia, and Chile are the main sending countries [10]. The flow of thousands of nurses each year to other countries negatively impacts health institutions in their home countries. Furthermore, nurse migration is a growing trend that is starting to impact historically nonexporter countries. For example, Mexican nurses express great desire to find labor opportunities abroad, specifically in Canada and the U.S., as populations in these countries are aging and require higher levels of care [18]. These countries are eager to receive nurses trained in Mexico in order to strengthen the capacity of their health systems and provide health services for elderly and retired populations [18]. Some analysts state that the migrant nursing workforce is concentrated on caring for aging populations, while U.S.- and Canadian-born nurses often work in clinical areas where salaries are higher [19]. Also, the growth of Spanish-speaking population in the U.S. (the minority group with the highest growth rate in the U.S.) attracts Mexican and other Latin American nurses, as compared to nurses from other developing countries.

## 4. Results

### 4.1. Nursing Personnel in the Americas

Nurses make up a significant proportion of the public health workforce in the Americas. While there are no data available about the number of public health nurses working in this region, there are separate data for the number of public health workers and the number of nurses.

It was only possible to identify the number of public health workers for seven countries, but their level of professional training could not be determined. It is important to note that Brazil has a significantly high number of public health personnel for every 10,000 inhabitants, surpassing Canada, Honduras, and Paraguay (see Table 2) [20–22].

The availability of nurses in the region is heterogeneous and it is not possible to establish a pattern of distribution according to economic indicators. In contrast to the availability of doctors, differences in the number of nursing personnel across the Americas do not correspond directly to level of economic development [18]. Puerto Rico reports 65.6 nurses per 10,000 people, while Jamaica reports 16.5. Other countries report notably lower ratios of nurses to inhabitants, such as Argentina (3.8), Chile (4.3), and Paraguay (2.8). In contrast, Canada, Cuba, and the U.S. report much higher ratios (see Table 3).

### 4.2. Literature Review

Only nine articles were identified that contained the search terms and met the inclusion criteria of the literature review. Results of the literature review suggest that although there is a range of articles about various topics pertaining to public health nursing, systematized information about the current state of the field across the Americas is unavailable (see Table 4). Reasons for the lack of organized information about public health nursing in the Americas include the use of varying names to refer to the field and the fact that few countries have established guidelines about or specific organizations dedicated to the field, among others. Also, the development of the field of public health nursing has varied across the Americas according to specific local, regional, and national health system demands, which are influenced by culture, history, politics, and economics; this increases the difficulty of finding systematized information about the profile of the public health nursing workforce in the Americas.

Public health nurses are referred to by a variety of titles in the Americas. Health professionals in the U.S. and Canada often use “public health nurse” or “community health practice.” Names used in Latin America and the Caribbean include the following: public health nurse (“enfermera de salud pública”) in Mexico, community health nurse (“enfermera de salud comunitaria) in Honduras; primary care health nurse (“enfermera de atención primaria de salud”) in Venezuela; visiting doctor (“visitador médico”) in Guyana; primary care psychiatric nurse (“enfermera de atención primaria psiquiátrica”) in Belize; rural nurse (“enfermera rural”) in Chile, family nurse (“enfermera familiar”) in Cuba; public health nurse (“enfermera sanitaria”); community nurse (“enfermera comunitaria”) [23]. Regardless of the name, this type of nursing generally refers to the area of health practice that normally occurs outside of hospitals, where the majority of nurses work.

A 2001 PAHO report defines a public health nurse as a nurse who has at least completed high school and works with a program or activity targeting population health, a public health organization, or an entity contracted with the government [8]. Despite this definition, the report states that many nurses unknowingly contribute to carrying out the essential public health functions and achieving health outcomes on a daily basis, even though they may not be familiar with terminology used in public health. For example, nurses often educate patients about healthy lifestyles and work with community groups to develop preventive health campaigns at the population level. Also, although nurses are often not recognized as responding to public health needs, in small communities and neighborhoods a public health nurse is often the only visible representative of the local public health system and acts as a first responder in cases of natural disasters and epidemics [8]. Because nurses are regularly in close proximity to patients—often more than doctors or other health authorities—their socially-perceived primary function is close, confidential, face-to-face patient contact, but the tasks that nurses perform in public health are diverse, ranging from one-on-one consultation with individuals and families to participation in community health campaigns.

In her article about nursing in Colombia, Gaviria-Noreña argues nurses should participate in health promotion and disease prevention through community participation and communication with individuals and groups, gathering socio-demographic data to monitor population health, and making decisions to restructure health services according to context-specific social, cultural, political, and economic factors [24]. While this explanation of nursing tasks does not use the term public health nursing, these responsibilities assigned to nurses are closely aligned with carrying out the essential functions of public health, demonstrating that the socially-perceived and practical roles of nursing and public health nursing intersect and overlap.

In addition, the PAHO report states that the classification of public health nursing as a separate field within the discipline of nursing is becoming less common, as health organizations tend to emphasize particular areas of health, such as vaccinations or maternal and child health, rather than public health more generally [8]. Despite the tendency to not use terminology that links nurses to public health, it is clear that nurses frequently work at the community and population levels to fulfill the essential functions of public health according to context-specific needs. For instance, public health nurses in Brazil focus on children with disabilities and hypertension, while those in Cuba participate in health promotion projects in workplaces and neighborhoods [8]. Although the report was published almost ten years ago, a consensus has yet to be achieved across the Americas regarding the definition, educational requirements, competencies, and workplace responsibilities of public health nurses and nurses who perform public health functions.

The Cuban nursing experience reported by Torres Esperón and Urbina Laza shows that changes in the National Health System in the 1980s resulted in the expansion of nursing job responsibilities to include community and population level actions [25]. These responsibilities include improving population health through outreach projects that promote healthy lifestyles and hygiene practices, and developing research that targets population health needs. Interestingly, Cuban nurses have a high level of representation at the governmental level due to the creation of the National Nursing Administration, which allows nurses to have more influence in National Health System decision-making. Thus, nurses in Cuba carry out a wide range of important public health functions, including providing care for individuals, families, and communities, performing health administration duties, training other nursing and health personnel, researching population health problems, and shaping health policy. Academic programs for all levels of nursing education and training—from auxiliary to specialist nurses—have significant curricular content concerning public health.

In Canada, the field of public health nursing has experiences changes in recent decades. Public health nurses have historically provided postnatal care and services, child health assessments, health promotion education in schools, immunizations, and community development projects associated with health issues [5]. Because the Canadian Health Act, established in 1984, defines essential health care mostly in terms of primary care services, provincial governments are largely responsible for disease prevention and promotion activities and campaigns, which are usually performed by community health organizations. Results of a 1999 survey of public health nurses in senior positions in Ontario and across Canada demonstrated that traditional public health nursing services offered to families and communities had diminished and significant regional disparities existed in regard to the availability of public health nurses [26]. The authors argue that changes in health policy and reductions in resources and funding are some of the causes for these changes in public health nursing. In their 2005 report, Armstrong-Stassen and Cameron state that in Ontario, public health nurses “focus on health promotion and disease prevention in a wide range of settings, including workplaces, schools, community centers, and other community agencies” and made up about 30% of the province’s community nurses [27]. Because these nurses work in such diverse environments, they must organize their work schedules to meet the demands of large caseloads, complex family and community related problems, while dealing with emotional stress and a lack of adequate resources and staffing.

The field of public health nursing is particularly well defined in the U.S. due to the sustained collaboration of academics and practitioners. The American Public Health Association defines public health nursing as “the practice of promoting and protecting the health of populations using knowledge from nursing, social and public health sciences” [28]. Due to changes in health policy and financing, these nurses must possess a range of skills in order to meet U.S. demand for medical services and population health promotion and prevention. In 2000, Gebbie and Hwang collaborated with health professionals from academia and state and local health agencies, including state nursing directors, to confirm the need for public health nursing training, based on the concept that public health nurses’ “principle focus is on populations or groups rather than individuals, regardless of the type of agency or organization that employed them...” Agencies could be private, voluntary, or non-official organizations or managed care entities [29]. While nurses employed in public health come from a range of educational backgrounds, the authors state that at the time of the research the only entry-level programs with curricula that address public health nursing theory and practice were baccalaureate programs. It is important to ensure that public health nurses possess a diverse set of skills, from epidemiological research to coalition building, but coordinating existing resources in order to guarantee that all nursing personnel acquire this knowledge is extremely difficult.

The Association of State and Territorial Directors of Nursing (ASTDN), which comprises a group of public health nursing leaders from across the U.S. and its territories, developed a set of public health nursing competencies in April 2003 [30]. The competencies were developed in collaboration with the Quad Council of Public Health Nursing Organizations, a group of four national nursing organizations: the Association of Community Health Nurse Educators, the American Nurses Association’s Congress on Nursing Practice and Economics, the American Public Health Association-Public Health Nursing Section, and ASTDN. Using as a starting point the “Core Competencies for Public Health Professionals,” developed by the Council on Linkages between Academia and Public Health Practice, ASTDN developed competencies specific to public health nursing at the generalist/staff and the manager/executive levels that enable nurses to strengthen local, regional, and national health infrastructure. Other reports provide similar competencies and standards for public health nursing practice, including the American Nurses Association [31] and nurses in the State of Georgia [32].

Cross *et al.* report that although many versions of public health competencies exist, including the aforementioned public health nursing competencies, is it difficult to measure changes in public health nursing competency levels [33]. In order to determine changes in public health nursing competency before and after continuing education and training, and the competency level of the public health nursing workforce overall, the authors developed an instrument comprising more than 195 measurable activities that correspond to the framework of the nursing process. Although the instrument’s duration and detail may limit its usefulness in certain public health settings, this instrument serves to clarify and demarcate the field of public health nursing.

Despite these advances, it is difficult to define the field of public health nursing in the Americas, and systematized information about health human resources across the region is difficult to obtain. Information is not available regarding the number and profile of health personnel directly involved in public health settings in Latin America and the Caribbean, especially in regard to nurses. Moreover, the established indicators used to measure the performance of public health professionals does not adequately account for the contributions of nurses [8]. PAHO provides information regarding the number of human resources from national sources in the Americas, but individual countries use different methods to gather health sector data. Moreover, local, regional, and national health systems across the Americas employ varying definitions of the role of nursing in public health systems. In contrast to those who define public health nursing by elaborating and refining established public health competencies, some experts argue that public health nursing should be examined from a perspective of nursing rather than public health, as these nurses provide a distinctive perspective gained from experiences in nursing education and the delivery of health services [7].

All of the sources consulted suggest that with the proper training and support public health nurses can significantly contribute to strengthening public health services, increasing the quality of the essential functions of public health, and improving the health of populations across the Americas. The results of the literature review suggest that more research is needed to determine how public health nursing competency impacts public health outcomes in the short and long terms and more attention must be given to nursing professors, as they must achieve competency in the essential functions of public health and nursing in order to comprehensively prepare students for future careers in public health nursing. Additionally, in spite of the substantial and diverse contributions of nurses to public health, it is important to note that their potential role in shaping health policy is often unacknowledged and requires future development, as they can provide critical insight about health at the individual, community, and population levels. Despite the similarities amongst the reviewed articles, definitions of public health nursing continue to differ in theory and practice and current information about the profile of public health nurses is not available for many countries in the Americas.

### 4.3. Description of Three Cases

The purpose of the description of the three cases is to explore the recognition of public health nurses in the labor market and issues regarding nursing training in Mexico, Colombia, and Paraguay, as well as to provide examples of how nurses can assume leadership roles to execute public health programs. The information gathered through the interviews supports what was found in the literature review. The information in this section comes from the interviewees’ comments, as well as official documents and grey literature provided by the interviewees.

#### 4.3.1. Mexico [34]

In Mexican nursing education, particularly at the high school level, programs of study include one semester of public health and three or more semesters of chemistry or math-related subjects. Some study plans include courses such as community nursing, and the majority address health education and teaching techniques. These topics are also common at the bachelor’s degree level. During high school level nursing training, practical experience gained through units about primary care is very limited, as programs of study prioritize units about hospital care.

A proposal to create a bachelor’s degree in public health nursing was developed by Mexican nursing societies and presented to the Inter-Institutional Commission for Human Resources for Health (ICHRH), a public agency coordinated by the Ministry of Health with the participation of universities, nursing schools, and health institutions. The Nursing Commission within ICHRH opposed this proposal with the argument that the labor market is not ready to demand nursing graduates from this type of program. However, the Commission recognizes the need to train more nurses with public health specialization at the graduate level.

According to information provided by the Secretary of Health, in 2007 there were 223,081 nurses in the public sector labor market, of which 209,907 had patient contact and 27,220 were specialists (see Table 5) [35]. Most of the nurses were general nurses who had completed high school and/or a bachelor’s degree.

The Nursing Commission of the Ministry of Health performed a survey in 2008 of 192,563 nurses and found that only 4,438 (or 2.3%) had studied public health in some way (see Table 6) [34].

Practically all nurses at the primary care level, who are predominantly auxiliary nurses, perform health promotion and prevention activities. A significant amount of their time is dedicated to developing national health programs, such as reproductive health, infant malnutrition, tuberculosis, vaccinations, and cancer. In the future, the participation of these nurses in monitoring individuals with chronic degenerative diseases and pregnant women should be strengthened. The prevention program of the Mexican Institute of Social Security (IMSS) offers training for its nursing personnel, which allows these professionals to develop their skills. The strength of the IMSS is based on the contributions of nursing personnel, as they provide information about health prevention and promotion for people insured by the IMSS. For example, nursing personnel give presentations and play videos about health prevention and promotion in waiting rooms at health centers and hospitals. They also offer individual consultation about health promotion and prevention in primary care settings. Furthermore, they provide periodic checkups for individuals with risk factors.

According to the professional job descriptions and work systems of the main employers in the public sector, specialist nurses who have completed graduate studies do not work in primary care. Nurses who have studied public health and have high school and bachelor’s degree level education are able to work in primary care.

Following this information about public health nursing in Mexico, it is possible to highlight the following:

- In nursing education at the high school level, there are few courses related to public health.
- Experience with primary care is very limited during training.
- The majority of nurses who perform public health tasks in Mexico are auxiliary and general nurses who work in primary care.
- Specialty nurses who have completed graduate education work exclusively in hospital units.
- The percentage of nurses who have explicitly studied public health is very low.

#### 4.3.2. Colombia [36]

According to the estimations of the University of Antioquia, Faculty of Public Health, in Colombia in 2005 there were 27,034 licensed nurses and 86,000 auxiliary nurses. Of these nurses, 23% had university training and 77% were auxiliary nurses [36]. Nurses with university training frequently hold positions within health institutions that allow them to address questions related to management, supervision, and planning, while auxiliary nurses work in direct contact with patients.

With respect to nursing training, there is a tendency of students to enroll in programs for auxiliary nurses with relatively basic educational requirements. Universities have not been able to substantially improve the nursing programs they offer, or increase the completion rate of students. The University of Antioquia is the only institution which offers a bachelor’s degree in nursing.

According to information provided by the Ministry of Social Protection, in the area of public health 12% of nursing professionals work at the community level. Interestingly, the main actors responsible for public health interventions are local mayors, as they manage the budgets to finance these activities. With the recent health system reform which began in 1993, there has been a significant decrease of nursing personnel who work at the community level with special programs related to prevention, promotion, and control of chronic and infectious health problems. This area of nursing has practically disappeared, but recently there has been interest in reinitiating community level strategies through a new vision of primary care. The nursing department at the University of Antioquia has become a leader in this area by evaluating the resurgence of indicators concerning tuberculosis, acute respiratory infections, and acute diarrheal illnesses of children.

During the past five years, the Nursing Department of University of Antioquia revisited primary health care as a strategy to implement a departmental development plan. This was achieved by identifying primary health care as a priority for general policy. This pilot strategy is noteworthy because it situates nursing personnel—from hospital-based nurses to nursing professors—as leaders of projects focused on family care. The program has a methodology of epidemiological surveillance combined with a socially sensitive design that targets families and the community, so that the population can learn about health promotion and disease prevention. There is a wide range of actions that involve health personnel, including providing consultation, monitoring disease, and preventing chronic problems, such as hypertension and diabetes. Other nurses lead health promotion projects that target youth to prevent anorexia, bulimia, sexually transmitted diseases, and teen pregnancy, which is a significant problem at the national level. Nurses also participate in projects with an explicit gender focus, especially those concerning reproductive health and domestic violence prevention.

#### 4.3.3. Paraguay [37]

Nursing education has a long history in Paraguay. In fact, the National University of Asunción began awarding bachelor’s degrees in nursing more than 45 years ago. Asunción, the capital of Paraguay, has the highest concentration of nurses with university training [38].

In general, nursing students are required to take public health courses during the four years of the degree program, and the majority of schools strongly emphasize public health in nursing education. Some of the major problems related to nursing education include the lack of a unified curriculum across programs, the lack of an evaluation and monitoring system for schools, and the shortage of well-trained nursing educators, especially at the auxiliary and technical levels.

In Paraguay, there are more than 20,000 nurses, of which 16.7% have earned a bachelor’s degree in nursing (see Table 7). In comparison with other countries in the region, Paraguay has one of the lowest ratios of nurses per 10,000 inhabitants (2.8), while Uruguay has 10.2, the Dominican Republic has 3.9, and Jamaica has 16.5 [39].

The Ministry of Public Health and Social Welfare employs 9,106 nurses, which represents 44.5% of Paraguay’s total nursing personnel. Of the nurses employed by the Ministry, 65.3% are auxiliary nurses (see Table 8).

All the nurses employed by the Ministry of Health perform public health tasks—from bachelor’s degree nurses to auxiliary and technical nurses. These nursing professionals focus on monitoring public health and managing health programs for immunizations, sexual and reproductive health, family planning, tuberculosis, diabetes, and epidemiological surveillance. Nursing personnel in the auxiliary and technical categories are able to assume responsibility of program execution, perform home visits, and give educational talks.

According to the Paraguayan Association of Nursing, recently nurses have received better working conditions and higher salaries. In fact, in some cases the salaries have doubled. Recently, the health sector renewed funding for 5,300 permanent positions, 2,900 of which are in the area of nursing. The creation of positions with fixed working schedules has increased the number of nurses. Also, the creation of a code of ethics and nursing law has strengthened the field of nursing.

## 5. Conclusions

- Across the Americas over the last 20 years, public health nurses have been growing in numbers and gaining recognition, despite the low position of public health as a priority area of health systems performance.
- The majority of nurses in Latin America have low-level qualifications in comparison with other groups of health professionals, such as doctors and dentists.
- According to the results presented in this study, in many countries in the Americas efforts to provide the appropriate personnel for public health activities have not been adequate.
- The definitions of public health nursing competencies and responsibilities are often aligned with national health systems reforms, which currently tend to focus on strengthening primary care and public health interventions.
- Generally, technical and auxiliary nurses perform public health tasks while nurses with specialist and university training work in clinical care in hospitals settings.
- The bibliographic search shows that journals that publish articles on public health nursing issues are scarce in the region. Only seven journals were identified in Canada, the U.S., Mexico, Colombia, Cuba, and Paraguay.
- More than 50% of the literature consulted was written by researchers from academic institutions and the rest by international consultants and governmental officials. This suggests that despite the scarcity of available information, a broad range of stakeholders have demonstrated interest in the field of public health nursing.
- The bibliographic search also suggests that there have been attempts to specify public health nursing competencies across the Americas, and the greatest advances have been made in Canada, the U.S., and Cuba.
- The definition of the 11 essential functions of public health has been useful for defining the limits of the field, but has not significantly changed the field of public health nursing in many countries in the Americas.
- It is important that countries in the region prioritize the development of the public health nursing workforce by focusing on training and defining the responsibilities of nurses in relation to public health services. These countries should also develop health system goals that include training human resources in primary care with a strong emphasis on collective interventions for population health.
- Due to the lack of information about nurses and their participation in the field of public health, it is only possible to outline some aspects of the field of public health and highlight areas that require future study.

## 6. Limitations of the Study

This study is limited by the scarcity of information available about public health nursing, as many sources provide only tangential or indirect references to the field. This study was also limited by the terms set for the wide-document search. Using broader search terms could have yielded more information about public health nursing. This study was also limited by the use of references in English and Spanish. Future research that uses French and Portuguese could provide more information about the region, such as the status of public health nursing in Quebec, Brazil, and Haiti.

## Notes Added in Proof

It is important to note that while the bibliographic search of this paper showed that journals that publish articles on public health nursing issues are scarce in the region, *Public Health Nursing* and the *Journal of Community Health Nursing* are dedicated to the field and thus offer multiple studies of the field in each issue.

In many countries in the Americas, public health nursing competencies and responsibilities support national health systems reforms, which currently center on improving primary care and public health interventions. This is not true for all countries in the Americas. For example, in the U.S., the American Nursing Association’s *Public Health Nursing: Scope and Standards of Practice* (2007)—an acclaimed publication in the field—clearly defines public health nursing and is not aligned with national health systems reforms. It is important to acknowledge the participation of the Association of Community Health Nurse Educators (ACHNE) in development of the publication.

It is also important to note that the Quad Council public health nursing competencies were developed as a joint effort by the four members of the organization: ACHNE, the American Nurses Association’s Congress on Nursing Practice and Economics (ANA), the American Public Health Association-Public Health Nursing Section (APHA), and the Association of State and Territorial Directors of Nursing (ASTDN).

It is only possible to outline some aspects of public health nursing in the Americas due to the lack of information about nurses and their participation in the field of public health in many countries. Reports published by U.S. organizations that focus on public health nursing issues offer clear explanations of the field, and could potentially serve as guides for future developments in the field of public health nursing in other countries.

Future research should focus on the impact of the field of public health nursing on population health.

## Figures and Tables

**Figure 1.**
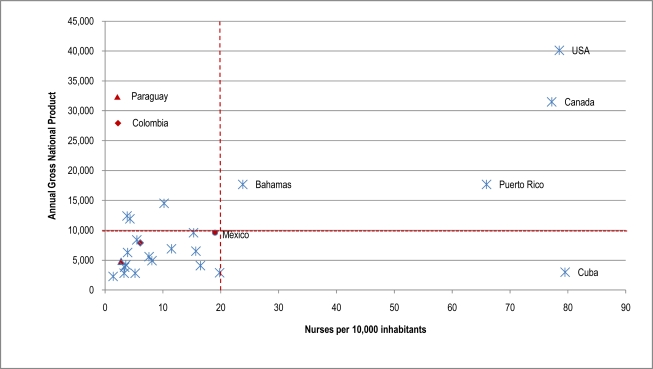
Nurses per 10,000 inhabitants in the Americas, 2005. Source: WHO. *Health Situation in the Americas: Basic Indicators*. WHO/PAHO: Washington, DC, 2008. Annual Gross National Product: http://www.photius.com/rankings/economy/gdp_per_capita_2005_0.html. (*) Includes only nurses that have completed a bachelor’s degree. Note: Information about the number of nurses is not available for the following countries: Anguilla, Antigua y Barbuda, Antilles, Aruba, Barbados, Bermuda, Bolivia, Dominica, Granada, Guadalupe, French Guyana, Cayman Islands, Turks and Caicos Islands, Virgin Islands (U.S.), Virgin Islands (UK), Martinique, Montserrat, Saint Kitts and Nevis, Santa Lucia, Suriname, Trinidad y Tobago, and Venezuela.

**Table 1.** Process to identify keywords.

	**Question**	**Concepts/Keywords**
Step 1	Is public health nursing a recognized and defined concept and/or field in the regional literature?	public health nursing, *enfermería en salud pública*, The Americas, *Las Américas*
Step 2	What are the perceptions and definitions of public health nursing in the region?	Latin America, *América Latina*, United States, *Estados Unidos*, Canada, *Canadá*
Step 3	What are the main topics in the fields of public health and nursing that are discussed in the regional literature?	public health, *salud pública*, nursing, *enfermería*
Step 4	What are the job responsibilities of nurses that are related to public health?	promotion, *promoción*, prevention, *prevención*
Step 5	What are the educational levels and types of training of nurses working in public health?	Education, *formación*, competencies, *competencias*

**Table 2.** Total and ratio of public health personnel for every 10,000 inhabitants.

**Country**	**Total Population**	**Public Health Personnel**	**Ratio for 10,000 Inhabitants**	**Year**
Brazil	170,115,000	167,080	10	2000
Canada	32,577,000	1,375	<1.0	2006
Costa Rica	4,023,000	1,266	3	2000
Honduras	6,485,000	215	<1.0	2000
Panama	2,856,000	948	3	2000
Paraguay	5,800,000	133	<1.0	2002
Saint Kitts and Nevis	38,000	17	4	2000

Sources: United Nations Population Division. *Population in 1999 and 2000: all countries*. UNPD: New York, NY, 2000.

United Nations Population Fund. *The State of World Population 2002: Demographic, Social and Economic Indicators*. UNFPA: New York, NY, 2002, 1–84.

WHO. *Statistical Information System (WHOSIS)*, 2006. http://www.who.int/whosis/en/ (accessed on September 7, 2009).

**Table 3.** Essential Functions of Public Health.

	Essential Functions of Public Health
1.	Monitoring, evaluation, and analysis of health status
2.	Surveillance, research, and control of the risks and threats to public health
3.	Health promotion
4.	Social participation in health
5.	Development of policies and institutional capacity for public health planning and management
6.	Strengthening of public health regulation and enforcement capacity
7.	Evaluation and promotion of equitable access to necessary health services
8.	Human resources development and training in public health
9.	Quality assurance in personal and population-based health services
10.	Research in public health
11.	Reduction of the impact of emergencies and disasters in health

Source: Ramagem, C.; Ruales, J. *The Essential Public Health Functions as a Strategy for Improving Overall Health Systems Performance: Trends and Challenges since the Public Health in the Americas Initiative, 2000−2007*. PAHO/WHO: Washington, D.C. February 2008.

**Table 4.** Summary of select articles about public health nursing.

**Author and Affiliation**	**Year**	**Title**	**Country/Region of Focus**	**Theme**
Land, S., World Health Organization, PAHO [23]	1998	*Enfermería Comunitaria en América Latina y el Caribe*	Latin America and the Caribbean	Diversity of community and public health nursing projects
Gebbie, K.; Hwang, I., Center for Health Policy and Health Services Research, Columbia University School of Nursing, New York, NY [29]	2000	Preparing Currently Employed Public Health Nurses for Changes in the Health System	U.S.	Education of Public Health Nurses
Gaviria-Noreña, D.L., Antioquia University, Nursing Faculty [24]	2000	*Modelo de Participación de Enfermería en Promoción de la Salud y Prevención de la Enfermedad*	Colombia	Nurse responsibilities to carry out essential public health functions
PAHO, Organization and Management of Health Systems and Services Program, Division of Health Systems and Services Development [8]	2001	*La Enfermería de Salud Pública y las Funciones Esenciales de Salud Pública*	The Americas	Relationship of public health nurses and the essential functions of public health
ASTDN, Quad Council of Public Health Nursing Organizations [30]	2003	Quad Council PHN Competencies	U.S.	Competencies for public health nursing
Armstrong-Stassen, M.; Cameron, S.J., University of Windsor [27]	2005	Concerns, Satisfaction, and Retention of Canadian Community Health Nurses	Canada	Job statisfaction and retention of community health nurses in Ontario
Falk-Rafael, A., York University; Fox, J., Simcoe County District Health Unit, Barrie, Ontario; Bewick, D., Family Health Services, Middle-Sex London Health Unit, London, Ontario [26]	2005	Report of a 1999 Survey of Public Health Nurses: Is Public Health Restructuring in Ontario, Canada Moving Toward Primary Health Care?	Canada	Changes in public health nursing practices in the Province of Ontario
Cross, S.; Bock, D.; Josten, L.; Reckinger, D.; Keller, L.; Strohschein, S.; et. al., St. Paul-Ramsey County Department of Public Health, Little Canada, Minnesota [33]	2006	Development of the Public Health Nursing Competency Instrument	U.S.	Instrument to measure public health nursing competencies
Torres Esperón, J.M., Omayada Urbina Laza [25]	2008	*La Enfermería en la Salud Pública Cubana*	Cuba	History of nursing and health sector reforms in Cuba

**Table 5.** Total and percentage of nurses in Mexico by category in the public sector, 1999–2007.*

**Categories**	**1999**	**%**	**2007**	**%**
Auxiliary	67,887	36.8	73,285	32.8
General	75,116	40.7	99,008	44.4
Specialist	23,641	12.9	27,229	12.3
Other	17,620	9.6	23,559	10.5
**Total**	**184,264**	**100**	**223,081**	**100**

* Includes interns and personnel in other positions without patient contact. Source: Secretary of Health, Statistical Information Bulletin, Volume 1, 1999 and 2007.

**Table 6.** Nurses with public health studies, 2009.

**Education Level**	**Total**
High School	3,659
Bachelor’s	579
Master’s	192
Doctorate	8
**Total**	**4,438**

Source: Interview with the directors of the Nursing Commission of the Secretary of Health (June 19, 2009).

**Table 7.** National total of registered nurses, 2009.

**Levels**	**Total**	**%**
Auxiliary	14,878	72.7
Technical	2,175	10.6
Bachelor’s Degree	3,405	16.7
**Total**	**20,458**	**100**

Source: Ministry of Public Health and Social Welfare of Paraguay, Department of Nursing, Partial Database, 2009.

**Table 8.** Nurses at the Ministry of Public Health and Social Welfare, 2009.

**Levels**	**Total**	**%**
Auxiliary in nursing	5,950	65.3
Technical	741	8.2
Bachelor’s Degree	2,415	26.5
**Total**	**9,106**	**100**

Source: Ministry of Public Health and Social Welfare of Paraguay, Department of Nursing, Partial Database, 2009.

## Appendix. Guide for Semi-Structured Interviews with Key Informants in Mexico, Colombia and Paraguay

Guide for Semi-Structured Interviews with Key Informants in Mexico, Peru, Colombia and Paraguay The objective of this interview is to determine the number of nursing personnel working in the Americas and describe the relationship of their work to public health, taking into consideration their professional training and the specific tasks they perform in the workplace. This information will be used to develop an article about public health nursing in the Americas.

1. What is the total number of nurses in your country (most recent data)?
2. How many nurses work in the public sector?
3. What categories of nurses exist (auxiliary, technical, general, specialists)? What level of training is required for each category?
4. How many nurses have direct contact with patients in primary care settings?
5. Of the nursing personnel who work in primary care settings, how many are trained in public health and at what level (high school, undergraduate, graduate)?
6. What public health tasks are performed by nurses?
7. What is the importance of nurses in public health?
8. What problems have been identified in regard to the relationships between training, nurses, and public health?
